# Ezrin contributes to cervical cancer progression through induction of epithelial-mesenchymal transition

**DOI:** 10.18632/oncotarget.7779

**Published:** 2016-02-27

**Authors:** Jienan Kong, Chunchan Di, Junjie Piao, Jie Sun, Longzhe Han, Liyan Chen, Guanghai Yan, Zhenhua Lin

**Affiliations:** ^1^ Department of Pathology and Cancer Research Center, Yanbian University Medical College, Yanji 133002, China; ^2^ Department of Pathology, The First Affiliated Hospital of Dalian Medical University, Dalian 116011, China; ^3^ Department of Pathology, Zibo First Hospital, Zibo 255200, China; ^4^ Department of Pathology, Yanbian University Hospital, Yanji 133000, China

**Keywords:** Ezrin, epithelial-mesenchymal transition, invasion, metastasis, uterine cervical cancer

## Abstract

Cervical cancer is the third most common cancer in females worldwide. The treatment options for advanced cervical cancer are limited, leading to high mortality. Ezrin is a membrane-cytoskeleton-binding protein recently reported to act as a tumor promoter, and we previously indicated that the aberrant localization and overexpression of Ezrin could be an independent effective biomarker for prognostic evaluation of cervical cancers. In this study, we identified Ezrin as a regulator of epithelial-mesenchymal transition (EMT) and metastasis in cervical cancer. Ezrin knock-down inhibited anchorage-independent growth, cell migration, and invasion of cervical cancer cell lines *in vitro* and *in vivo*. EMT was inhibited in Ezrin-depleted cells, with up-regulation of E-cadherin and Cytokeratin-18 (CK-18) and down-regulation of mesenchymal markers. Ezrin knock-down also induced Akt phosphorylation. These results implicate Ezrin as an EMT regulator and tumor promoter in cervical cancer, and down-regulation of Ezrin suppressed cervical cancer progression, possibly via the phosphoinositide 3-kinase/Akt pathway. Furthermore, the expression pattern of Ezrin protein was closely related with the lymphovascular invasion status of cervical cancer by immunohistochemistry, and the survival analysis revealed that the cervical cancer patients with the perinuclear Ezrin expression pattern had longer survival time than those with the cytoplasmic Ezrin expression pattern. Ezrin thus represents a promising target for the development of novel and effective strategies aimed at preventing the progression of cervical cancer.

## INTRODUCTION

Cervical cancer is the third most common cancer in females worldwide. Advanced cervical cancer is associated with a high mortality because of the limited treatment options, with over 85% of patients dying in developing countries [1]. Patients diagnosed with early-stage disease have good long-term survival after treatment [2], but the 5-year survival rates for patients with stage III and IV disease remain < 40%. Moreover, approximately 30% of patients experience lymph node recurrence and distant metastases after primary treatment [3]. The application of molecular target drugs has resulted in improved overall survival and recurrence rates in several human malignancies [4]. However, no target drugs for cervical cancer have yet been identified. Elucidation of the molecular mechanisms underlying the metastasis and invasion of cervical cancer is therefore crucial for improving its prognosis.

Ezrin is encoded by the EZR gene located at chromosome 6q25.2-q26. It is a membrane-cytoskeleton-binding protein and a member of the ezrin/radixin/moesin protein family [5]. Li *et al.* found that Ezrin silencing using small hairpin RNA reversed the metastatic behavior of human breast cancer cells [6], indicating an important role for Ezrin in regulating tumor metastasis and progression. The results of several studies suggest that Ezrin may play a key role in tumor development, invasion, and metastasis, probably through regulation of adhesion molecules, participation in cell signal transduction, and signaling to other cell membrane channels in the tumor [7–11]. We recently reported that Ezrin was over-expressed in cervical cancer, and its expression was closely related to metastasis and poor prognosis.

Importantly, Saito *et al.* found that Ezrin suppression induced the expression of E-cadherin and β-catenin in the HSC-3 tongue squamous cell carcinoma cell line [12], indicating a possible correlation between Ezrin and cadherin on switching of EMT. EMT is considered to be a crucial step in the progression of most carcinomas. During EMT, the actin cytoskeleton is reorganized and cell-matrix contacts are increased, leading to dissociation from the surrounding cells and enhanced migratory and invasive capabilities [13]. Determination of the mechanisms governing EMT is therefore essential for the development of novel therapeutic strategies to overcome cancer metastasis [14]. Thus, Ezrin may function as metastasis-related oncogene by modulating multiple cellular processes including maintenance of cell shape, cell-cell adhesion, and cell motility and invasion [8, 15–18]. However, the mechanisms whereby cervical cancer cells acquire the ability to invade nearby tissues and metastasize, and how Ezrin activates EMT in cervical cancer are poorly understood.

In this study, we determined the expression of Ezrin in primary cervical cancer tissues and cervical cancer cell lines, including HeLa, SiHa, CaSki and C33A, and also explored the correlation of its expression with EMT markers. Additionally, we clarified the role of Ezrin in cervical cancer progression by silencing its expression by RNA interference (RNAi). We therefore defined specific oncogenic activities of Ezrin in cervical cancer both *in vitro* and *in vivo*, and identified the molecular mechanisms whereby Ezrin contributes to cancer invasion and metastasis. The results suggest that Ezrin may represent a novel therapeutic target for the reversal of EMT and for preventing metastasis in cervical cancer.

## RESULTS

### Ezrin expression in cervical cancer cells and tissues

**Figure 1:**
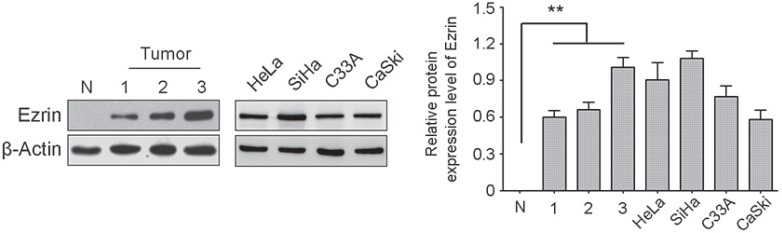
Ezrin protein expression level was determined in cervical cancer cells and tissues by western blot Ezrin was highly expressed in four human cervical cancer cell lines and three primary cervical cancers. Actin was used as a loading control. The blot was over-exposed to emphasize low expression in normal tissue (N).

We detected Ezrin protein expression levels in HeLa, SiHa, C33A, and CaSki cervical cancer cell lines and in three cervical cancer and one normal cervical tissue samples using western blot (Figure 1). Ezrin protein was highly expressed in all the cervical cancer cell lines, and was significantly increased in cervical cancer tissues compared with the normal tissue sample. In previous study [5], we found that Ezrin expression was significantly higher in cervical cancers than in CIN, CGIN, and normal cervical epithelia. Ezrin overexpression was closely related with poor differentiation, late stage, and lymph node metastasis, suggesting that Ezrin is frequently over-expressed in cervical cancer.

### Ezrin is successfuly knocked down in HeLa and SiHa cells

**Figure 2:**
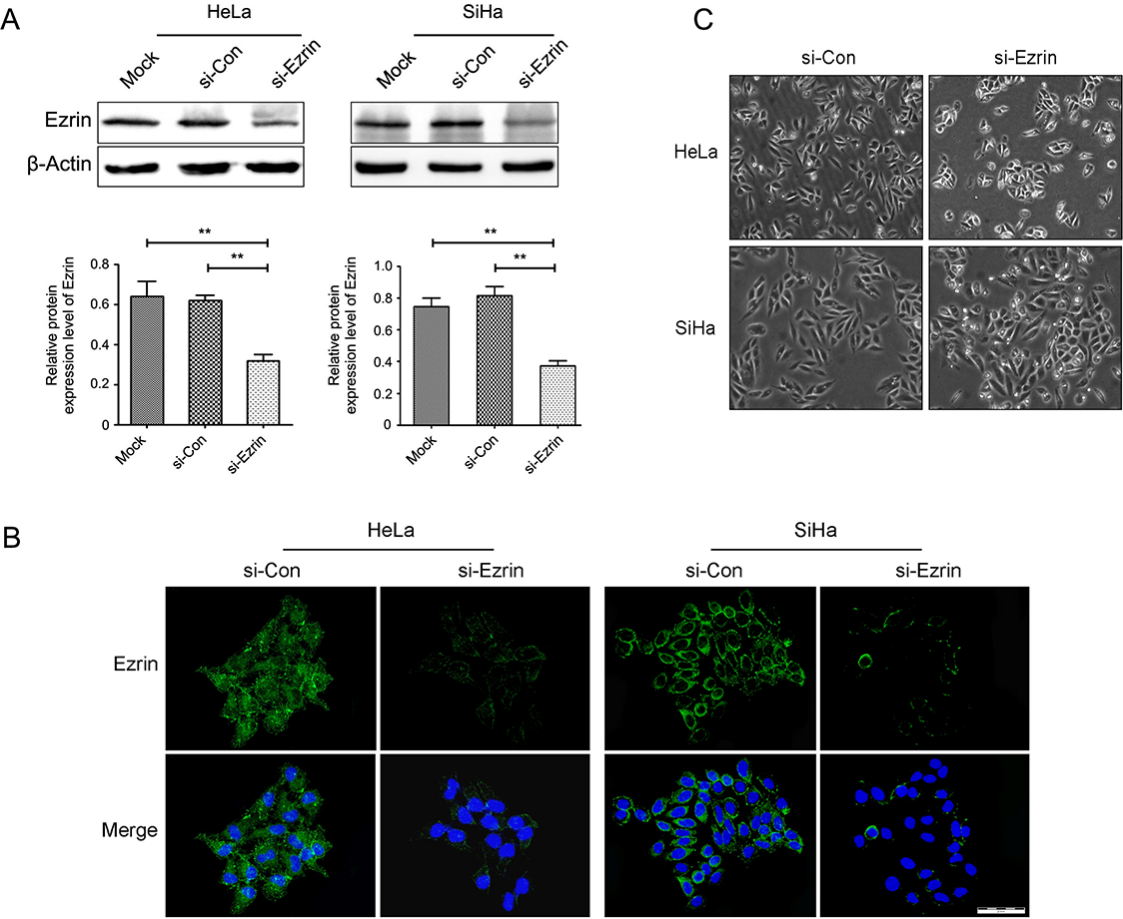
Ezrin was knocked down by siRNA in cervical cancer cells by western blot and IF staining (**A**) Ezrin protein expression was inhibited in si-Ezrin-transfected HeLa and SiHa cells compared with si-control cells, as demonstrated by western blot (*P* < 0.05). (**B**) Cytoplasmic Ezrin (green) protein expression was inhibited in si-Ezrin-transfected HeLa and SiHa cells compared with si-control cells, as demonstrated by IF staining. DAPI staining (blue) was included to visualize the nucleus. (**C**) Ezrin-depleted cells increased cell-cell contacts compared with control cells.

We knocked down Ezrin expression in both HeLa and SiHa cells using si-Ezrin transfection, and confirmed the knock-down effects by western blot and immunofluorescent (IF) staining. Ezrin expression was remarkably reduced in si-Ezrin transfected HeLa and SiHa cells compared with mock-transfected and untransfected controls, according to western blot and IF staining (Figure 2A and 2B). The IF staining also showed changes in cell morphology following si-Ezrin transfection, the control cells maintained a fibroblastic, elongated, mesenchymal-like morphology, while si-Ezrin transfected cells showed a round, cobblestone, epithelial-like phenotype, especially in SiHa cell (Figure 2C).

### Ezrin knock-down inhibits the proliferation and the ability of cell migration and invasion of HeLa and SiHa cells

**Figure 3:**
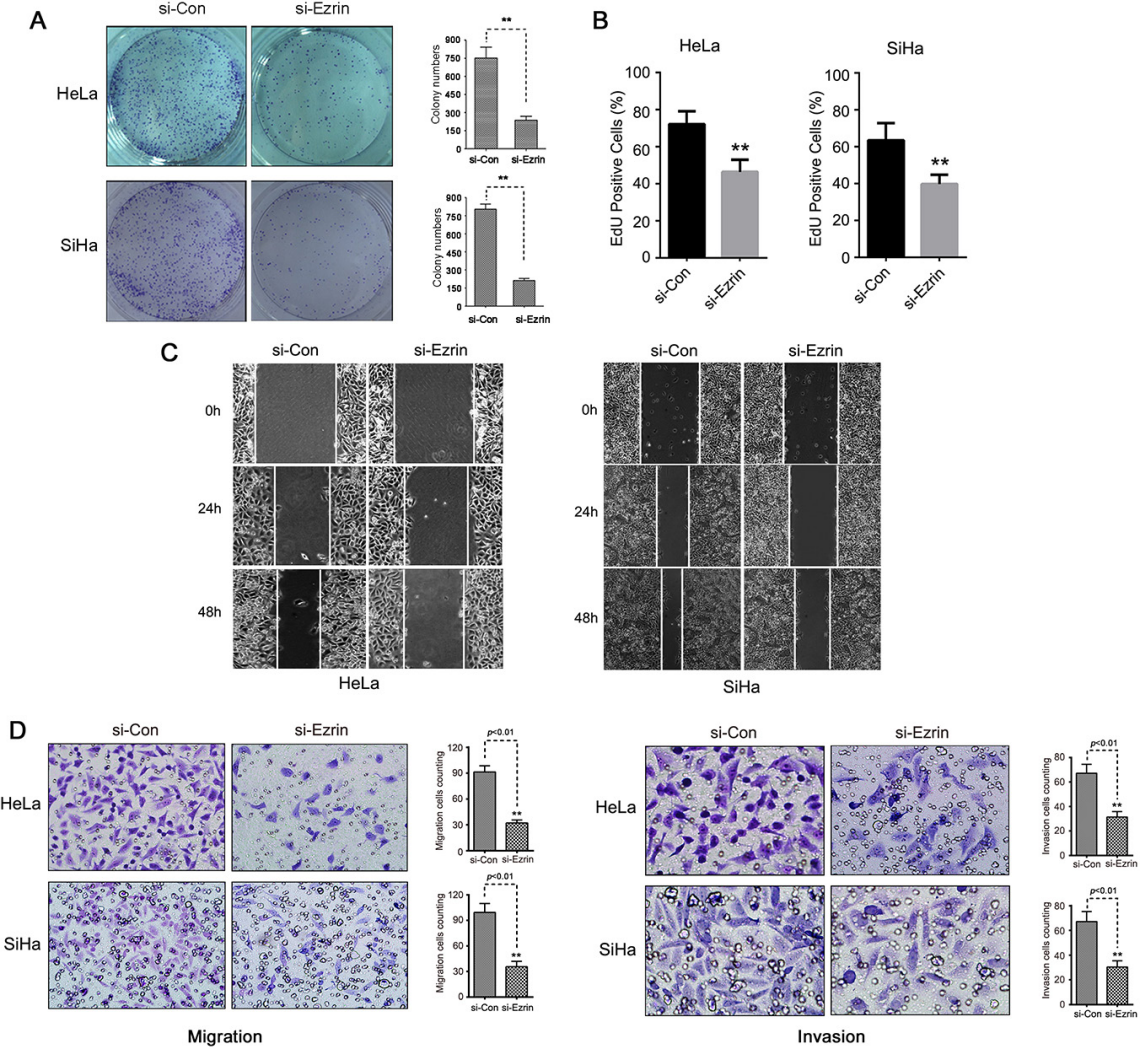
Ezrin knock-down decreased the growth and the ability of migration and invasion of cervical cancer cells *in vitro* (**A**) The number of colonies per plate was counted. Data are shown as mean ± standard deviation of at least three independent experiments. (**B**) Proliferating capability of transfected cells was evaluated using EdU incorporation. (**C**) Scratch wound-healing assay was used to determine the effects of si-Ezrin on HeLa and SiHa cell motilities. (**D**) Migration of Ezrin knock-down cells was measured by transwell migration assay. The invasive abilities of HeLa and SiHa cells were determined in Boyden chamber assay after transfection with si-Ezrin or si-control. The results were quantitated by counting invading cells in five randomly chosen high-power fields for each replicate. *P* values were obtained using *t*-tests.

We firstly determined the effects of Ezrin knock-down (KD) on cell proliferation using colony-formation assay, and found that the cells transfected with si-Ezrin formed fewer colonies than the cells transfected with si-control (Figure 3A). Then, the EdU analysis was used to confirm the inhibitory ability of Ezrin depletion in both HeLa and SiHa cells, the results indicated that the cell proliferation was reduced after Ezrin KD (Figure 3B). Thus, Ezrin KD could effectively inhibit the growth and the proliferation rate of cervical cancer cells *in vitro*. We then investigated the role of Ezrin in cervical cancer cell motility and invasion by wound-healing and transwell assays. Ezrin KD reduced the migration of HeLa and SiHa cells after 24 and 48 h compared with si-control cells, as determined by wound-healing assay (Figure 3C). Ezrin KD was also associated with significantly reduced migration and invasion abilities in both HeLa and SiHa cells in transwell assay (Figure 3D). These results indicate that Ezrin depletion increased cell-cell contacts and suppressed cell proliferation, oncogenic transformation, and migration and invasion potentials, suggesting an important role of Ezrin in cervical cancer progression.

### Effects of Ezrin KD on EMT in cervical cancer cells *in vitro*

**Figure 4:**
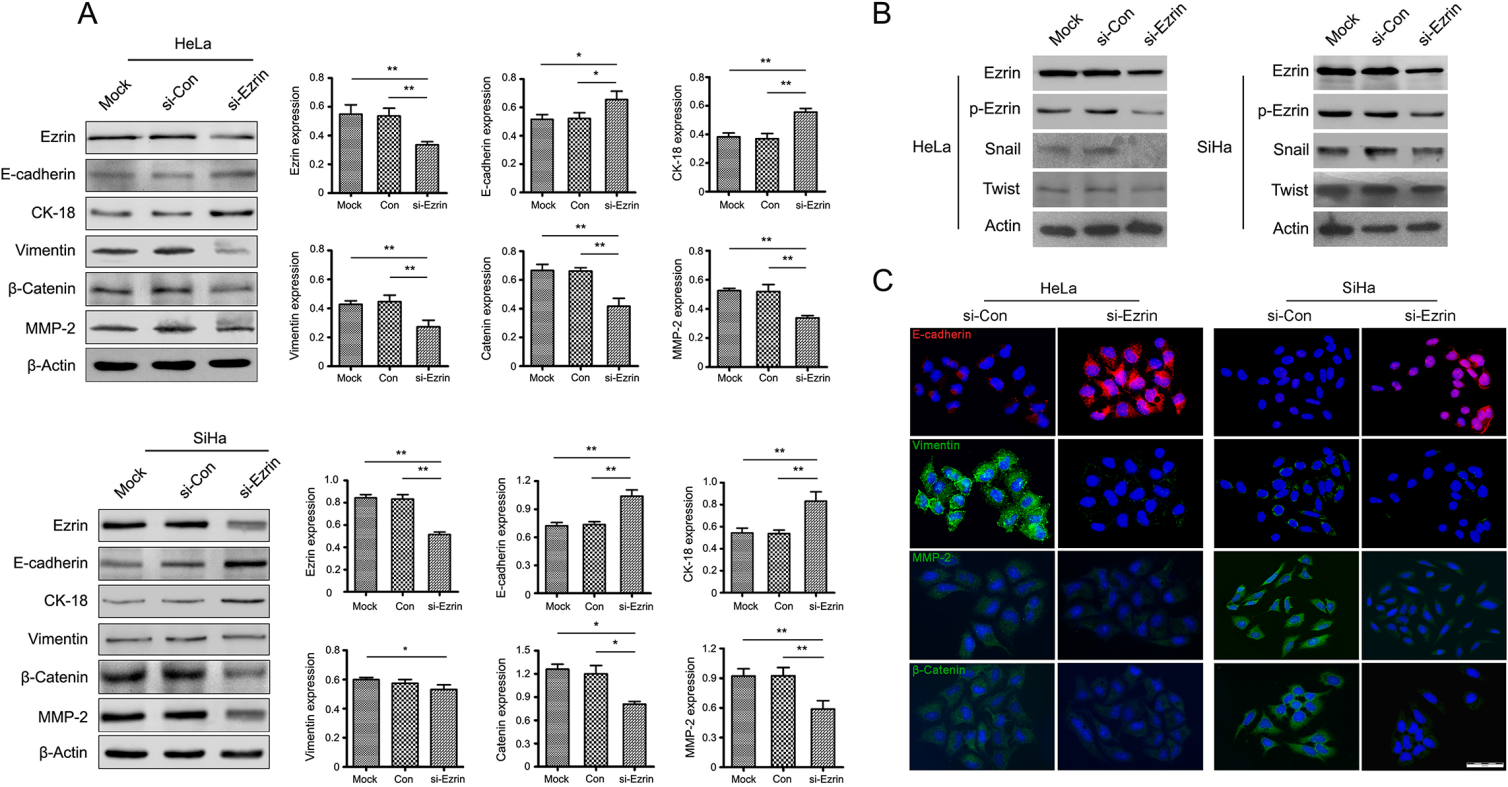
Ezrin regulates EMT in cervical cancer (**A**) Western blot analysis of EMT markers in HeLa and SiHa cells transfect with si-Ezrin and si-control. β-Actin was used as a loading control. (**B**) Western blot analysis of Ezrin, *p*-Ezrin, transcription factor Snail and Twist expressed in si-Ezrin and si-control cells. (**C**) Expression of EMT markers was detected by IF staining in HeLa and SiHa cells transfect with si-Ezrin and si-control.

Enhanced cell migration and invasion are important consequences of EMT. Ezrin KD decreased cell-cell contacts and significantly reduced cell migration and invasion. We therefore hypothesized that Ezrin may play an important role in the EMT process. Morphologic and phenotypic EMT-like changes are accompanied by down-regulation of the epithelial marker E-cadherin and up-regulation of mesenchymal markers such as Vimentin and matrix metalloproteinase-2 (MMP-2). The expression levels of EMT markers were detected by western blot. Epithelial markers, including E-cadherin and CK-18, were significantly increased, while the mesenchymal markers Vimentin, MMP-2 and β-catenin were decreased, and transcription factors Snail, Twist were also decreased in si-Ezrin-transfected cells compared with si-control-transfected (Figure 4A and 4B).

**Figure 5:**
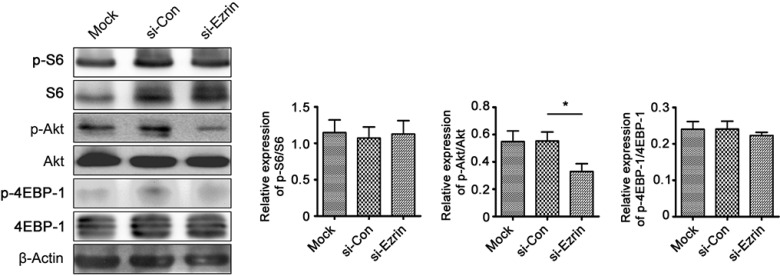
Effects of Ezrin KD on Akt phosphorylation, determined by western blot Akt phosphorylation was decreased in si-Ezrin-transfected HeLa cells. β-Actin was used as a loading control.

We also examined the expression levels and localization of epithelial and mesenchymal markers in si-Ezrin and si-control transfected cells using IF staining. Consistent with the results of western blot, Ezrin KD significantly increased E-cadherin expression and decreased Vimentin, MMP-2, and β-catenin expression in HeLa and SiHa cells (Figure 4C), suggesting that Ezrin KD inhibited EMT in cervical cancer cells *in vitro*. Furthermore, we investigated the signaling mechanisms involved in the Ezrin-mediated EMT process by determining the effects of Ezrin depletion on the activities of Akt, S6 and 4EBP-1. Ezrin depletion reduced Akt phosphorylation, while total Akt levels remained unchanged (Figure 5, *P* < 0.05). This suggests that Ezrin promotes EMT, at least in part through up-regulation of phosphoinositide 3-kinase (PI3K)/Akt signaling pathways.

### Effects of Ezrin on invasion ability of cervical cancer cells *in vivo*

**Figure 6:**
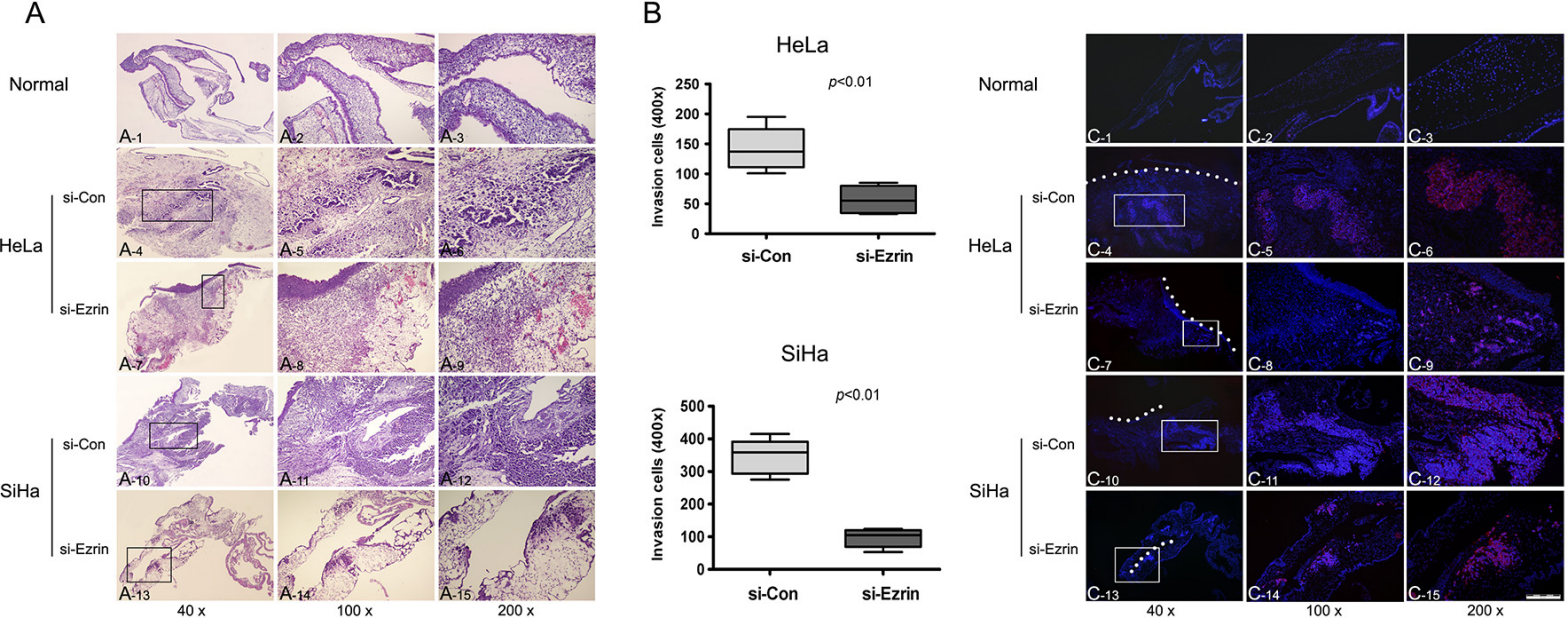
Invasion of cervical cancer cells in the chick chorioallantoic membrane (CAM) by H & E and IF staining (**A**) (A_1_–A_3_) Normal structure of CAM layers; (A_4_–A_9_) HeLa cells transfected with si-Ezrin showed less invasion into the CAM than si-control-transfected cells; (A_10_–A_15_) SiHa cells transfected with si-Ezrin showed less invasion into the CAM than si-control-transfected cells. (**B**) Quantification of the invasion assay was shown in the panels, respectively. Each column represents the mean of five different fields. (**C**) CAM sections were stained with DAPI for basement membrane (blue) and the CAM surface is marked by dashed white lines, and cervical cancer cells were stained with p16 antibody (red). (C_1_–C_3_) Normal structure of CAM layers; (C_4_–C_9_) HeLa cells transfected with si-Ezrin showed less invasion into the CAM than si-control-transfected cells; (C_10_–C_15_) SiHa cells transfected with si-Ezrin showed less invasion into the CAM than si-control-transfected cells.

We observed the role of Ezrin in cancer cell invasion by chorioallantoic membrane (CAM) invasion assay using hematoxylin and eosin (H & E) and IF staining. Cells were cultured on top of the chick CAM. After culture for 3 days, si-control-transfected HeLa and SiHa cells spread rapidly across the CAM surface and infiltrated the underlying stroma (Figure 6). The invasion was dramatically inhibited by Ezrin KD.

### The significance of Ezrin expression pattern in association with the lymphovascular invasion and survival status in cervical cancers

**Figure 7:**
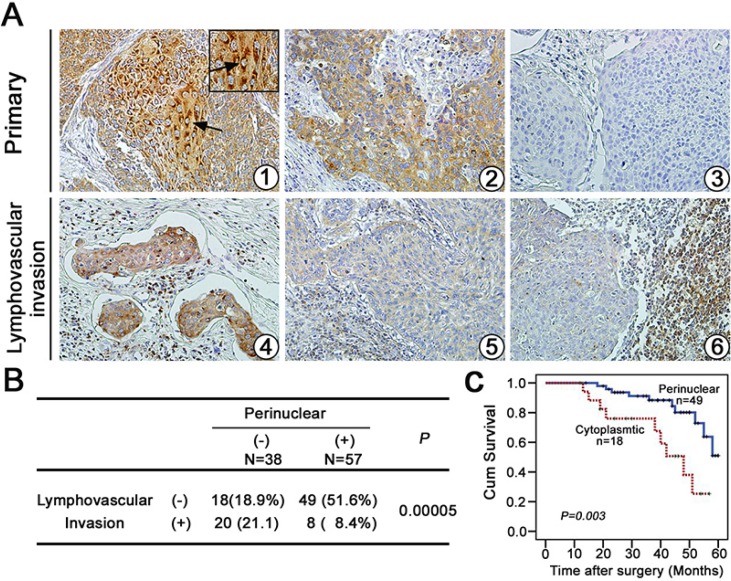
The perinuclear expression type of Ezrin protein with survival analysis in cervical cancers (**A**) IHC analysis of Ezrin expression in primary cervical cancer (① ∼ ③) and matched tissues (④ ∼ ⑥) with lymphovascular invasion (×200). (**B**) Perinuclear expression pattern of Ezrin protein was closely related to lymphovascular invasion status in cervical cancer. (**C**) Kaplan-Meier survival analysis showed that the overall survival of patients with perinuclear Ezrin expression pattern was significantly better than that of patients with the cytoplasmic staining pattern of Ezrin.

Metastasis is a multistep process including local invasion, intravasation, transport, extravasation, and colonization by which tumor cells disseminate from their primary site and form secondary tumors at a distant site [21]. In previous study, we found that Ezrin expression was significantly higher in cervical cancers than in normal cervical epithelia. Ezrin over-expression was closely related with lymph node metastasis. Furthermore, the positive rate of perinuclear localization of Ezrin was significantly higher in early stage cervical cancers compared with advanced stage cases (*P* < 0.05), demonstrating that the localization of Ezrin might be significantly associated with differentiation and stage in cervical cancer [5]. Here we further assessed the significance of Ezrin expression pattern in 95 cases of cervical cancers, which showed positive staining of Ezrin protein using IHC, and found that the perinuclear expression pattern of Ezrin protein was significantly related with the lymphovascular invasion status in cervical cancers (Figure 7A and 7B). Additionally, as shown in Figure 7C, Kaplan-Meier survival analysis showed that the overall survival of patients with perinuclear Ezrin expression was significantly better than that of patients with the cytoplasmic staining pattern of Ezrin (*P* = 0.003), suggesting that perinuclear Ezrin expression may predict a longer survival and negative lymphovascular invasion in patients with cervical cancer.

## DISCUSSION

Despite improvements in diagnostic and screening techniques and the availability of vaccines, cervical cancer remains the second largest cause of cancer-related deaths in women worldwide [22]. A good understanding of the mechanisms of metastasis and the identification of new targets are therefore prerequisites for improving therapies for cervical cancer.

Down-regulation of cell-cell contacts and increased cell motility and invasion are key steps in the metastatic cascade. Ezrin is an important membrane-cytoskeleton crosslinking protein known to stimulate several cytoskeleton-related functions. It has been implicated in many aspects of cancer cell biology and has been shown to participate in the regulation of cell shape, adhesion, motility, and apoptosis, and to correlate with invasion and metastasis in many types of human cancers [23, 24]. Ezrin expression has been found to be positively related to the degree of malignancy in many tumors, and its expression has also been linked to poor survival in several cancers, including carcinomas of the breast [25], endometrium [26] and in melanomas [27] and soft tissue sarcomas [28, 29]. Over-expression of Ezrin protein enhanced the metastatic potential of a variety of tumors including carcinomas of the endometrium [26, 30] and pancreas [31]. Ezrin also plays a crucial role in morphogenesis [32], and an absence of Ezrin was associated with morphological changes in cancer cells through actin cytoskeleton remodeling [12]. In accord with these results, we also observed the changes in cervical cancer cell morphology following Ezrin KD. There is increasing evidence to suggest that Ezrin regulates tumor progression. For example, Xie *et al.* and Li *et al.* reported that interference with Ezrin expression suppressed the growth, adhesion, and invasiveness of esophageal squamous cell carcinoma and lung cancer cells [33, 34].

Cell proliferation as a consequence of cell cycle progression leads to clonal expansion of cells during tumor promotion [35]. Zhang *et al.* indicated that decreasing Ezrin expression significantly inhibited cell proliferation, mobility, and invasiveness in hepatocellular carcinoma cell lines, and that Ezrin enhanced the growth of cancer cells by supporting cell division and cell cycle progression from G0/G1 to S and G2/M phases [36]. Saito *et al.* showed that Ezrin down-regulation increased the G0/G1 fraction but decreased the G2/M fraction by interfering with mitosis and cell cycle progression [12]. Consistent with these results, we found that cervical cancer cells transfected with si-Ezrin formed significantly fewer colonies and showed less proliferating ability than si-control (*P* < 0.05), suggesting that Ezrin KD inhibited the proliferation of cervical cancer cells. We further determined the role of Ezrin in cervical cancer cell motility by wound-healing assay. Compared with si-control cells, Ezrin KD markedly reduced the wound-healing abilities of both HeLa and SiHa cells, while migration and invasion assay confirmed that Ezrin was involved in the cell migration and invasion abilities of cervical cancer cells (*P* < 0.05).

Increasing evidence indicates an important role for Ezrin in the development and progression of malignant tumors. EMT is believed to be a critical step in the progression of neoplasms, by conferring migratory and invasive properties [37]. Malignant tumors develop via a complex process involving multiple factors and stages, and malfunctions or mutations of a variety of genes. Although the roles of Ezrin in tumor cell migration and invasion abilities are well understood, other Ezrin-mediated tumor cell activities remain to be elucidated, including its role in EMT. Li *et al.* suggested that Ezrin was important for the localization of E-cadherin to the plasma membrane and regulation of the c-Src/β-catenin/E-cadherin signaling pathway in breast cancer cells [6]. Moreover, Saito *et al.* hypothesized that Ezrin-induced down-regulation of E-cadherin and β-catenin may be associated with actin remodeling and the formation of podia extensions [12]. We examined the relationships between Ezrin expression and the expression of EMT-related markers to clarify the molecular mechanisms underlying these observations. Western blot and IF staining showed that Ezrin KD was associated with up-regulation of epithelial markers, such as E-cadherin and CK-18, and down-regulation of Vimentin, β-catenin, and MMP-2, compared with si-control. However, MMP-9 expression was unaffected by Ezrin KD (data not shown). For the localization of E-cadherin, Lohia *et al.* indicated that loss of Scrb causes E-cadherin accumulation into perinuclear vesicles [38]. Céspedes *et al.* and Elzagheid A *et al.* reported that the shift of E-cadherin localization from the membrane to the cytoplasm or nucleus was associated with metastasis and poor outcomes of patients with colorectal cancer [39, 40]. In current study, we found the increased membranous expression of E-cadherin in SiHa cells with Ezrin depletion, moreover, the increased expression of E-cadherin in both membrane and cytoplasm were also observed in HeLa cells with Ezrin depletion. Therefore, the mechanism by which the E-cadherin molecule is translocated to the cytoplasm and nucleus in the invasive cancer cells needs the further study to clarify.

**Figure 8:**
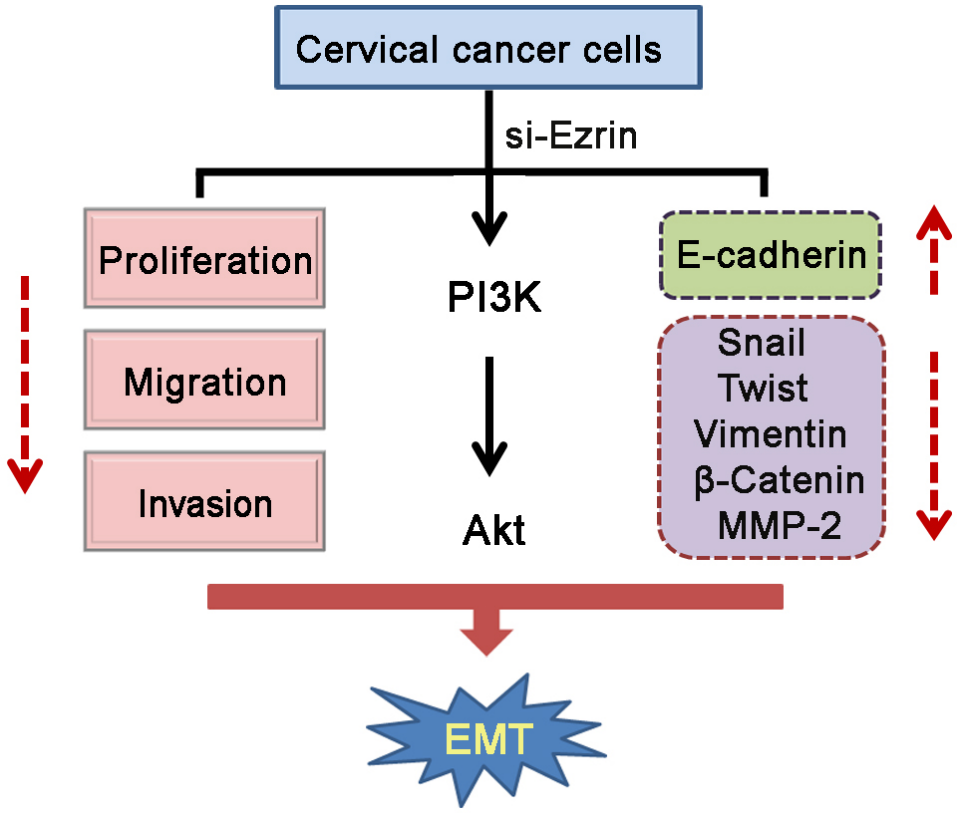
Diagram showing the model proposed for the function of silenced-Ezrin in cervical cancer cells

*In vivo* experiments also showed that Ezrin KD reduced the ability of cervical cancer cells to cross the CAM surface and infiltrate the underlying stroma. Overall, these studies suggest that Ezrin promotes cervical cancer cell proliferation, invasion, and metastasis through EMT (Figure 8).

Ezrin is involved not only in cytoskeletal organization, but also in several signaling pathways. Ezrin was shown to interact with neural cell adhesion molecule L1 and regulate the NF-κB signaling pathway in colon cancer [41]. Furthermore, Ezrin-mediated early metastasis was partially dependent on activation of the ERK/mitogen-activated protein kinase (MAPK) pathway in osteosarcoma [42], while depletion of Ezrin down-regulated the MAPK and transforming growth factor-β pathways in esophageal squamous cell carcinoma [33]. Ezrin also participates in the activation of MAPK and PI3K in both breast and prostate cancers [43]. Xie *et al.* revealed that ERK phosphorylation was reduced in Ezrin-KD cells, and activation of the ERK/MAPK pathway might partially attenuate Ezrin-mediated suppression of cell invasiveness in esophageal squamous cell carcinoma [33]. Ezrin-mediated effects on Akt and ERK1/2 activity have been linked to its ability to promote tumor progression and metastasis [9].

Previous experiments demonstrated that phosphorylation of Ezrin on residue Y145 promoted cell proliferation, whereas phosphorylation on residue Y353 activated the PI3K/Akt pathway, thus providing a mechanism for the role of Ezrin in cell survival [44]. However, Karishnan *et al.* revealed that Ezrin action depended on the Akt/mTOR signal transduction cascade, but not on MAPK in Ewing's sarcoma [45].

Ezrin over-expression could increase the activity of Akt through plasma membrane co-localization, resulting in decreased apoptosis via the mTOR signal transduction pathway [46]. Our results indicated that down-regulation of Ezrin decreased Akt phosphorylation (*P* < 0.05), but had no significant effect on ERK1/2 protein phosphorylation (*P* > 0.05), suggesting that the PI3K/Akt pathway might participate in Ezrin-mediated EMT in uterine cervical cancers. However, the further studies are needed to confirm these results. Additionally, Auvinen *et al.* reported that enhanced expression of Ezrin was observed in cervical HPV-associated lesions, suggesting Ezrin's role in the development of cervical cancer [47]. We also detected the expression levels of HPV E6 and E7 proteins in si-Ezrin and si-control HeLa and SiHa cells, and found that Ezrin dowregulation has no significantly impact on HPV E6 and E7 expressions (Figure S1). Thus, the further study is needed to verify the mechanism whether HPV mediates the progression of the EMT via Ezrin in cervical tumorigenesis.

In conclusion, Ezrin expression was successfully silenced in cervical cancer cells by RNAi and the down-regulation of Ezrin significantly inhibited proliferation, migration, and invasion of uterine cervical cancer cells through EMT inhibition. These effects may be related to the PI3K/Akt pathway. The perinuclear expression of Ezrin protein is significantly associated with the longer survival time of patients with cervical cancer. Ezrin thus represents a promising new target for the development of novel and effective strategies for preventing the progression of cervical cancer.

## MATERIALS AND METHODS

### Tissue specimens and cell lines

Routinely processed and diagnosed uterine cervical lesion tissues, including 95 squamous cell carcinomas (SCCs) of the uterine cervix, which were selected from the Department of Pathology and Tumor Tissue Bank, Yanbian University Medical College, and the cancer patients were aged 23–79 years. All the specimens were selected from hysterectomies between 1998 and 2012, and all data were retrieved from patients’ operative and pathological reports. Staging was performed according to the TNM and FIGO classification of carcinomas of the uterine cervix; 69 were considered early stage (FIGO stages I-IIA) and 26 advanced stage (IIB-IV); 29 were keratinizing and 66 non-keratinizing, according to the Union for International Cancer Control (UICC) criteria 7th Edition and WHO classification. A total 67 of cervical cancer patients had follow-up records for more than 5 years, and the follow-up deadline was December 2013. The survival time was counted from the date of surgery to the follow-up deadline, or date of death (usually the result of cancer recurrence or metastasis).

The human cervical cancer cell line HeLa, SiHa, C33A and CaSki were purchased from the ATCC. HeLa cells were cultured in DMEM (Gibco BRL, Grand Island, NY, USA) containing 10% fetal bovine serum (FBS) and 1% pen-strep (GIBCO BRL, USA). SiHa, C33A and CaSki were grown in RPMI-1640 medium containing 10% FBS and 1% pen-strep. The cell lines were cultured in a 37°C with 5% CO_2_ and were split twice a week.

### Antibodies

Antibodies against Ezrin, p-Ezrin, E-cadherin, Vimentin, Snail, p-S6, p-Akt, p-4EBP-1, S6, Akt, 4EBP-1 and β-Actin were purchased from Cell Signaling Technology (Boston, USA). Cytokeratin 18, HPV18 E6 and E7 antibody was purchased from Santa Cruz (Dallas, USA). Antibody against MMP-2 was purchased from BOSTER (Wuhan, China). MMP-9 and β-cantenin were purchased from Affinity (Cincinnati, USA). Antibody against Twist was purchased from Abcam (Cambridge, UK).

### Transfection

We purchased two different Ezrin siRNA, including si-RNA1 and si-RNA2, from RIBOBIO (China). According to the KD effect, si-RNA1 was used in this study. The sequence of si-RNA1 (si-Ezrin) was 5′-AAGGAAUCCUUAGCGAUGAGA-3′. Additionally, control siRNA (si-control) was also used in this study. Cells were transfected with 30 nM siRNA using Lipofectamine 3000 (Invitrogen) according to the manufacturer's instructions. For the KD effect of si-RNA2, we showed the some results in Figure S2.

### Morphology observation

HeLa and SiHa cells were cultured in media containing 10% fetal bovine serum at 37°C in a humidified atmosphere containing 5% CO_2_. A total of 2 × 10^5^ cells were plated in 6-well plate. When the cells reached 60–70%, cells were cultured in Opti-MEM. Cells were transfected with 30 nM siRNA using Lipofectamine 3000 (Invitrogen) according to the manufacturer's instructions. After 48 h, cells were taken out of the incubator and placed under a microscope.

### Wound healing assay

Cells were plated onto six-well plates at 95% confluence in complete tissue culture medium. After the cells became confluent, cell wounds were created by scratching cells using a micropipette tip. The medium was then immediately replaced, and spontaneous cell migration was monitored using a Nikon inverted microscope at 0 and 48 h. The distance of wound closure was measured in three independent wound sites per group. The wound gaps were measured at each time.

### Immunofluorescence

Cells grown in six-well culture slides fixed with 4% paraformaldehyde for 15 min, permeabilized with 0.5% Triton X-100 (CWBIO, China) and blocked with 3% BSA for 2 h. Cells were incubated with primary antibody in 3% BSA at 4°C overnight, washed three times with PBS, incubated with Alexa Fluor 488 or Alexa Fluor 546-labeled secondary antibody (Invitrogen) in 3% BSA for 2 h, and then analyzed by Leica SP5II confocal microscope.

### Cell migration assay

The migration assay used 24-well Millicell (MILLIPORE) with 8-μm PET. Cells were seeded into the upper insert in serum-free media, while media containing 10% FBS was added to the lower chambers as a chemoattractant for 12 h. The cells were removed from the upper surface of the filter by scraping with a cotton swab. Cells that infiltrated through the filter were fixed and stained with Gemisa. The images were taken with OLYMPUS BX53.

### Invasion assay

The invasion assays used 24-well BD BioCoat Matrigel invasion chambers with 8-mm pore inserts (BD). Cells (5 × 10^4^) suspended in serum-free media were seeded in the upper inserts, while media containing 10% FBS was added to the lower chambers as a chemoattractant. The cells were removed from the upper surface of the filter by scraping with a cotton swab after 48 h in culture. Cells that infiltrated through the filter were fixed and stained with Gemisa. The mean values of the results obtained with the three chambers were used in the analysis.

### Colony-forming assay

Single-cell suspension (1000 cells per well) were seeded in six-well plates and incubated for 2 weeks. The cells were fixed by ice-cold methanol and stained by Gemisa for 25 mins. Washing 30 mins with tap water, and the colonies (more than 50 cells) were counted directly on the plate. Statistical significant was calculated from each three independent experiments.

### EdU assay

Cell proliferation was determined by 5-ethynyl-2′-deoxyuridine (EdU) incorporation assay, which was carried out using Cell-Light^™^ EdU Apollo^®^488 *In Vitro* Imaging Kit (RiboBio) according to the manufacturer's instructions and analyzed by Leica SP5II confocal microscope.

### Western blot

Immuno-blot analysis was performed as previously described [19]. Briefly, cells were harvested and lysed with RIPA buffer containing 1 mM PMSF and a protease inhibitor cocktail (Roche, Germany). The protein concentration of each sample was measured using BCA protein assay kit (Pierce, Rockford, Illinois). Protein samples (20 μg/lane) were electrophoresed (Bio-Rad, Hercules, CA) on 8%–12% SDS polyacrylamide gel and transferred to PVDF membranes (Bio-Rad, USA) in a transfer buffer. Membranes were blocked by incubation in 5% skim-milk (diluted in Tris-buffered saline and 0.2% Tween-20) for 1.5 h, and probed with appropriate antibodies (1:1000) at 4°C for overnight followed by probing with second antibody goat anti-rabbit IgG-HRP and goat anti-mouse IgG-HRP diluted with TBST to 1:1000, and shake membrane at RT for 1.5 h. Detection by enzyme-linked chemiluminescence (ECL) was performed according to the manufacturer's protocol. Results were analyzed quantitatively using Chemiluminescent and Fluorescent Imaging System.

### Chick chorioallantoic membrane assays (CAM assay)

Fertilized chicken eggs were incubated in incubator at 37.8°C with 60–65% humidity. Ethics approval was obtained by the University of Yanbian Animal Ethics Committee. HeLa and SiHa cervical cancer cells (si-control, si-Ezrin) *in vivo* was assessed using 11-day-old chick embryos in which an artificial air sac was created under aseptic conditions [20]. A total of 1 × 10^6^ cells were inoculated atop the chick chorioallantoic membrane (CAM) and the window was resealed with adhesive tape and eggs were returned to the incubator until day 14 of chick embryo development. The CAM was removed at the surrounding CAM were harvested from each embryo and fixed with 4% paraformaldehyde for 24 h and embedded in paraffin. Serial sections (4 μm) were stained with H & E.

### Immunohistochemistry

For immunohistochemical studies with a DAKO LSAB kit (DAKO A/S, Glostrup, Denmark), 4 μm-thick tissue sections were deparaffinized, rehydrated and incubated with 3% H_2_O_2_ in methanol for 15 minutes at room temperature to eliminate endogenous peroxidase activity. Antigen retrieval was performed by placing the slides in 0.01 M sodium citrate buffer (pH 6.0) at 95°C for 20 minutes. After overnight incubation at 4°C with primary antibody against Ezrin (Cell Signaling, USA), sections were treated according to standard immunoperoxidase methods using a streptavidin-biotin peroxidase complex kit (LSAB+Kit/HRP, DAKO). The peroxidase reaction was developed with 3, 3′-diaminobenzidine (DAB), and then counterstained with Mayer's hematoxylin. Rabbit IgG isotope used as a negative control and positive tissue sections were processed omitting the primary antibody as a further negative control. All slides were evaluated independently by two pathologists without prior knowledge of clinical outcome.

### Statistical analysis

The data analysis was performed using SPSS 17.0 software and GraphPad Prism 6.0 software. Group comparisons for continuous data were done by *t*-test for independent means or one-way ANOVA. Survival curves were calculated using the Kaplan-Meier analysis. Biochemical experiments were performed in triplicate and a minimum of three independent experiments were evaluated. The value of *P* < 0.05 was considered statistically significant.

## SUPPLEMENTARY MATERIALS FIGURES


