# Supplementary material for: Ezrin contributes to cervical cancer progression through induction of epithelial-mesenchymal transition
Source: Oncotarget. 2016 Feb 27;7(15):19631–42. doi: 10.18632/oncotarget.7779 (PMC4991407; doi:10.18632/oncotarget.7779)
Supplement: Supplementary file 1 [file oncotarget-07-19631-s001.pdf]

Ezrin contributes to cervical cancer progression through induction of epithelial-mesenchymal transition

Supplementary Materials

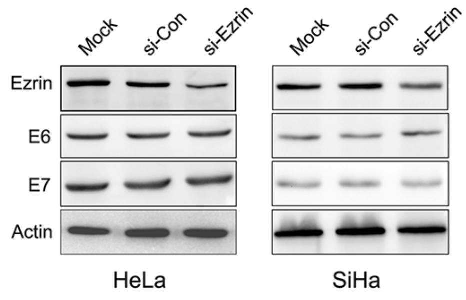

Supplementary Figure S1: Ezrin dowregulation has no significantly impact on HPV E6 and E7 expression levels. β-Actin was used as a loading control.

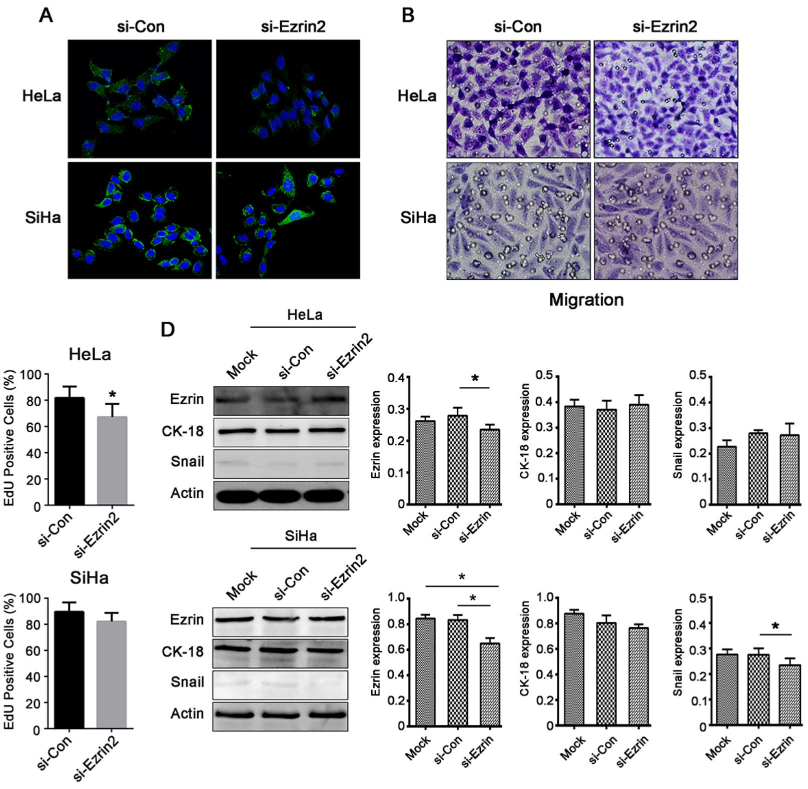

Supplementary Figure S2: Effects of cells transfected with si-Ezrin2 in HeLa and SiHa cells. (**A**) Cytoplasmic Ezrin (green) protein expression in HeLa cells transfected by si-Ezrin2 was decreased compared with the cells transfected by si-control by IF staining. However, the KD effects were not significant in SiHa cells. DAPI staining (blue) was included to visualize the nucleus. (**B**) Migration of Ezrin KD cells was measured by transwell migration assay. (**C**) Proliferating capability of transfected cells was evaluated using EdU incorporation. (**D**) Western blot analysis of EMT markers in HeLa and SiHa cells transfected with si-Ezrin2 or si-control, respectively. β-Actin was used as a loading control.
